# Обзор клинических рекомендаций по гипопаратиреозу

**DOI:** 10.14341/probl12800

**Published:** 2021-08-17

**Authors:** Е. В. Ковалева, А. К. Еремкина, Ю. А. Крупинова, С. С. Мирная, И. В. Ким, Н. С. Кузнецов, Е. Н. Андреева, Т. Л. Каронова, И. В. Крюкова, А. М. Мудунов, И. В. Слепцов, Г. А. Мельниченко, Н. Г. Мокрышева, И. И. Дедов

**Affiliations:** Национальный медицинский исследовательский центр эндокринологии; Национальный медицинский исследовательский центр эндокринологии; Национальный медицинский исследовательский центр эндокринологии; ООО "Сеть семейных медицинских центров №1"; Национальный медицинский исследовательский центр эндокринологии; Национальный медицинский исследовательский центр эндокринологии; Национальный медицинский исследовательский центр эндокринологии; Национальный медицинский исследовательский центр эндокринологии; Московский областной научно-исследовательский клинический институт им. М. Ф. Владимирского; Национальный медицинский исследовательский центр онкологии им. Н.Н. Блохина; Клиника высоких медицинских технологий им. Н.И. Пирогова Санкт-Петербургского государственного университета; Национальный медицинский исследовательский центр эндокринологии; Национальный медицинский исследовательский центр эндокринологии; Национальный медицинский исследовательский центр эндокринологии

**Keywords:** эпидемиология, гипопаратиреоз, паратиреоидный гормон, кальций, витамин D, терапия

## Abstract

Гипопаратиреоз — эндокринное заболевание, характеризующееся сниженной продукцией паратиреоидного гормона околощитовидными железами или резистентностью тканей к его действию, что сопровождается нарушениями фосфорно-кальциевого обмена.Основной этиологией гипопаратиреоза является повреждение или удаление околощитовидных желез во время хирургического вмешательства на органах шеи. Ввиду распространенности рака щитовидной железы, первичного гиперпаратиреоза и других патологий органов шеи, радикальное лечение которых может привести к развитию гипопаратиреоза, прогнозируется неуклонный рост и увеличение числа больных с этой патологией. Аутоиммунный гипопаратиреоз — вторая по распространенности форма заболевания, встречающаяся, как правило, в рамках аутоиммунного полигландулярного синдрома 1 типа. Гипопаратиреоз в рамках этого синдрома возникает в детском возрасте и, как правило, характеризуется более тяжелым течением, особенно в случае сопутствующего синдрома мальабсорбции.Развитие хронического гипопаратиреоза любой этиологии требует пожизненного назначения многокомпонентной терапии, а также тщательного мониторинга и индивидуального подхода к ведению заболевания. В отсутствие адекватного динамического наблюдения развиваются множественные осложнения со стороны жизненно важных органов, в частности кальцификация мочевыделительной системы (нефрокальциноз, нефролитиаз с развитием почечной недостаточности), мягких тканей и головного мозга; сердечно-сосудистые нарушения; зрительные расстройства; патология костно-мышечной системы со снижением костного ремоделирования и потенциальным риском переломов, а также развитием нейрокогнитивных расстройств и резким снижением качества жизни пациентов.Своевременная диагностика, рационально подобранная лекарственная терапия и грамотное ведение пациента позволят снизить риски развития осложнений, позволят улучшить прогноз, снизить частоту госпитализаций и инвалидизаций пациентов с данным заболеванием.В статье изложены основные тезисы клинических рекомендаций по ведению пациентов с гипопаратиреозом, утвержденных Минздравом России в 2021 г. Изложены алгоритмы диагностики, лечения и динамического наблюдения за пациентами с данной нозологией. Отдельные разделы посвящены купированию острой гипокальциемии, а также ведению беременности у пациенток с гипопаратиреозом.

## Abstract (en)

Hypoparathyroidism is a rare disorder characterized by the absent or inappropriately decreased serum parathyroid hormone in the parathyroid glands, which is accompanied by impaired calcium-phosphorus metabolism.The main etiology of hypoparathyroidism remains damage or removal of the parathyroid glands during neck surgery. In view of the incidence of thyroid cancer, primary hyperparathyroidism and other pathologies of the neck organs, which radical treatment can lead to the parathyroid gland impairment, an increased number of patients with hypoparathyroidism is expected. Autoimmune hypoparathyroidism is the second most common form of the disease, usually occurring as part of type 1 autoimmune polyglandular syndrome. Autoimmune hypoparathyroidism usually occurs in childhood and is characterized by a severe course of the disease, especially in the case of concomitant malabsorption syndrome.Chronic hypoparathyroidism of any etiology requires lifelong multicomponent therapy, as well as careful monitoring and an individual approach to choose the optimal treatment strategy. In the absence of adequate follow-up, the risks of long-term complications significantly increase, particularly in the renal, cardiovascular systems; in the soft tissues and in the brain, it could lead to visual disturbances; pathology of the musculoskeletal system with a decreased bone remodeling and a potential risk of fractures, as well as to the neurocognitive disorders and an impaired health-related quality of life.Timely diagnosis, rational medical therapy and management strategy may reduce the risks of short-term and long-term complications, frequency of hospitalizations and disability of patients, as well as improve the prognosis.This review covers the main issues of Russian guidelines for the management of chronic hypoparathyroidism, approved in 2021, including laboratory and instrumental evaluation, treatment approaches and follow-up. This guidelines also include the recommendations for special groups of patients: with acute hypocalcemia, hypoparathyroidism during pregnancy.

## ВВЕДЕНИЕ

Гипопаратиреоз — состояние, характеризующееся сниженной продукцией паратиреоидного гормона (ПТГ) околощитовидными железами (ОЩЖ) или резистентностью тканей к его действию, что сопровождается нарушениями фосфорно-кальциевого обмена.

При гипопаратиреозе отсутствие или недостаточность ПТГ неизбежно сопровождается развитием гипокальциемии. К основным патогенетическим механизмам относятся: снижение активности остеокластов с уменьшением высвобождения кальция из костей; повышение экскреции кальция с мочой; подавление синтеза кальцитриола в почках и снижение абсорбции кальция из кишечника. Дефицит ПТГ приводит к гиперфосфатемии как напрямую посредством увеличения почечной тубулярной реабсорбции фосфатов, так и косвенно за счет гипокальциемии. Хроническая гиперфосфатемия у пациентов с гипопаратиреозом, как было показано, ассоциирована с повышением в крови уровня фактора роста фибробластов 23.

## ЭТИОЛОГИЯ

Хирургическое вмешательство на органах шеи — самая распространенная причина развития гипопаратиреоза, обуславливающая около 75% всех случаев данного заболевания. Послеоперационный гипопаратиреоз может быть обусловлен как непосредственным удалением, так и интраоперационной травмой или нарушением кровоснабжения ОЩЖ. Риск хронического гипопаратиреоза тесно связан с количеством оставшихся in situ функционирующих ОЩЖ во время операции: 16% при сохраненных 1–2 железах, 6% — при 3 железах и 2,5% — при 4 [1][2].

Аутоиммунный гипопаратиреоз — вторая по распространенности форма гипопаратиреоза, обусловленная иммуноопосредованным разрушением клеток ОЩЖ [3]. Он может быть изолированным заболеванием, однако значительно чаще встречается в рамках наследственного аутоиммунного полигландулярного синдрома (АПС) 1-го типа [4]. АПС 1-го типа — моногенное аутосомно-рецессивное заболевание, в основе которого лежит нарушение структуры гена аутоиммунного регулятора (AIRE). В основе патогенеза заболевания лежит аутоиммунная деструкция различных эндокринных желез, включая ОЩЖ. Для АПС 1-го типа характерна классическая триада: слизисто-кожный кандидоз, гипопаратиреоз, хроническая надпочечниковая недостаточность. Заболевание дебютирует, как правило, в детском возрасте.

Другие более редкие наследственные формы гипопаратиреоза встречаются как в изолированном варианте, так и в составе поликомпонентных генетических заболеваний (синдром ДиДжорджи, Бараката, Кенни–Каффи и др.) [5–10].

В случае нарушения обмена магния развивается функциональный гипопаратиреоз, который является обратимой формой заболевания с восстановлением функции ОЩЖ после коррекции гипо-/гипермагниемии [5][11].

В редких случаях причиной гипопаратиреоза могут стать инфильтративные заболевания, такие как саркоидоз, амилоидоз, тиреоидит Риделя и метастатическое поражение ОЩЖ [12–15]. Ткань ОЩЖ относительно не восприимчива к лучевому повреждению, тем не менее в литературе описаны очень редкие случаи радиационно-индуцированного гипопаратиреоза [16–19]. Отложения минералов в ткани ОЩЖ — например, меди при болезни Вильсона и железа при гемохроматозе — являются редкими причинами развития гипопаратиреоза. Описаны случаи развития гипопаратиреоза вследствие массивных повторяющихся трансфузий у пациентов с талассемией [20–24].

## ЭПИДЕМИОЛОГИЯ

Гипопаратиреоз — это редкое заболевание с распространенностью 0,25 на 1000 населения. Имеющиеся данные о распространенности гипопаратиреоза основаны на крупных эпидемиологических исследованиях, проведенных в США, Дании, Норвегии и Италии. Полученные результаты относительно сходны и свидетельствуют о распространенности гипопаратиреоза в диапазоне 23–37 на 100 000 населения [3][25–28].

Послеоперационный гипопаратиреоз чаще встречается среди женщин, что связано с более частой патологией щитовидной железы и, следовательно, тиреоидэктомией [29][30]. Распространенность наследственных форм гипопаратиреоза не различается у мужчин и женщин [27]. В российской популяции крупных эпидемиологических исследований с целью оценки распространенности гипопаратиреоза не проводилось.

Классификация гипопаратиреоза [31].

* — случаев развития гипопаратиреоза в рамках аутоиммунного полигландулярного синдрома 2 типа (АПС 2 типа) не описано.

## ЖАЛОБЫ И КЛИНИЧЕСКАЯ КАРТИНА

Основные клинические проявления гипопаратиреоза обусловлены наличием гипокальциемии. Усиление чувствительности сенсорного (чувствительного) нейрона проявляется в виде парестезий в конечностях и в околоротовой области; моторного (двигательного) нейрона — мышечными спазмами, вплоть до тетании; от классического карпопедального спазма до жизнеугрожающего ларингоспазма [32]. Тяжелая гипокальциемия ассоциирована как с локальными, так и генерализованными судорогами тонико-клонического типа.

Степень выраженности симптомов зависит от уровня кальция в сыворотке крови, а также от скорости прогрессирования гипокальциемии. Для хронического течения заболевания характерна адаптация к низким уровням кальция сыворотки крови с отсутствием выраженной клинической картины даже при тяжелой гипокальциемии. Провоцирующими факторами ухудшения состояния в таких случаях могут являться возрастание физической активности, медицинские процедуры, беременность и лактация [16][33].

Самой частой жалобой пациентов с гипопаратиреозом является наличие судорог и/или парестезий в мышцах верхних и нижних конечностей, околоротовой области. Пациенты с длительным анамнезом гипопаратиреоза предъявляют жалобы на «мозговой туман», снижение памяти и концентрации внимания. Основные жалобы пациентов с гипопаратиреозом представлены в табл. 1.

Клинические проявления хронического гипопаратиреоза различны и затрагивают почти все системы организма (табл. 2). Клинические симптомы хронического гипопаратиреоза могут быть ассоциированы как с эпизодами гипо-, так и гиперкальциемии, гиперфосфатемии [16].

**Table:** Таблица 1. Симптомы гипопаратиреоза [34]

Симптом	Частота, %
Физические симптомы
Усталость	82
Боль в мышцах/мышечные спазмы	78
Парестезии	76
Тетания	70
Боли в костях и суставах	67
Расстройства кишечника	46
Хрупкость/ломкость ногтей	44
Непереносимость жары	44
Головные боли	42
Сухость кожи и ее повышенная травматизация	40
Зябкость	37
Выпадение волос	33
Тошнота	30
Проблемы с зубами	29
Нейропатия	27
Чувствительность к солнцу	26
Отеки	23
Проблемы с дыханием	22
Снижение слуха	11
Когнитивные симптомы
«Мозговой туман»/умственная летаргия	72
Невозможность концентрировать внимание	65
Снижение памяти/забывчивость	61
Нарушение сна	57
Эмоциональные симптомы
Тревожность/страх/внутреннее беспокойство	59
Снижение настроения/грусть/депрессия	53
Эмоциональная чувствительность	47
Социальная изоляция	32

**Table:** Таблица 2. Клинические симптомы гипопаратиреоза

Системы органов	Клинические проявления
Периферическая нервная система	Парестезии, фибриллярные подергивания, тонические судороги, тетания, карпопедальный спазм, симптомы Хвостека и Труссо
Центральная нервная система	Невроз, снижение памяти, бессонница, депрессия
Дыхательная система	Ларингоспазм и бронхоспазм
Желудочно-кишечный тракт	Дисфагия, рвота, диарея и запоры
Сердечно-сосудистая система	Удлинение интервала Q–T и неспецифические изменения зубца T, дилатационная гипокальциемическая кардиомиопатия
Почки	Нефролитиаз/нефрокальциноз, снижение функции почек
Скелетно-мышечная система и зубы	Миопатия скелетных мышц, спондилоартропатия, гипоплазия зубной эмали, укорочение корней, гипоплазия или отсутствие зубов
Органы зрения	Субкапсулярная катаракта, папиллоэдема
Кожные покровы	Сухость кожи, хрупкость ногтей, онихолизис, пустулезный псориаз

## ЖАЛОБЫ И АНАМНЕЗ

Комментарии. Следующие факторы позволяют заподозрить у пациента наличие гипокальциемии и гипопаратиреоза:

- проведение хирургического вмешательства в области шеи;

- наличие парестезий в области лица, верхних и нижних конечностей;

- наличие фибриллярных подергиваний отдельных мышц, судорог в проксимальных мышцах;

- выявление кальцификации головного мозга;

- при нарушениях сердечного ритма.

## ФИЗИКАЛЬНОЕ ОБСЛЕДОВАНИЕ

Комментарии. Положительный симптом Труссо — появление судорог в кисти («рука акушера») через 1–3 минуты после сдавления плеча манжетой при измерении артериального давления. Данный симптом — высокочувствительный и специфичный признак гипокальциемии — выявляется у 94% пациентов с гипокальциемией и у 1% людей с нормокальциемией. Cимптом Хвостека — сокращение мышц лица при постукивании в месте выхода лицевого нерва — менее чувствительный и специфичный признак. Отрицательный симптом Хвостека наблюдается у 30% пациентов, имеющих гипокальциемию, положительный — у 10% людей без данной патологии (рис. 1).

**Figure:**
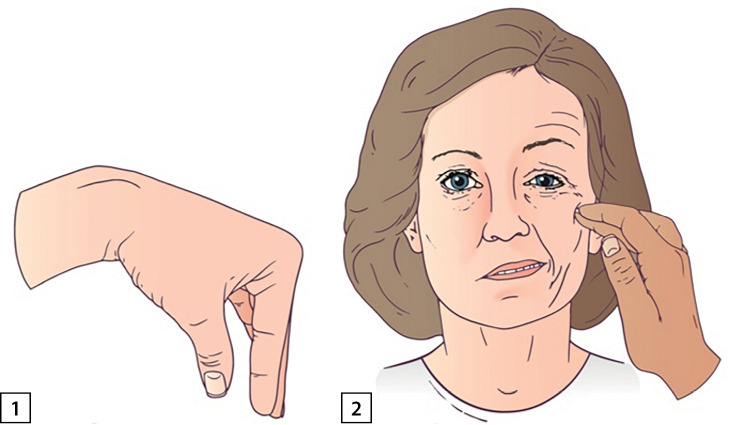
Рисунок 1. 1 — проба Труссо, «рука акушера»; 2 — симптом Хвостека.

Диагноз гипопаратиреоза основывается на результатах лабораторного обследования!

Критерии установления диагноза гипопаратиреоз.

Комментарии. Наиболее распространенным тестом для диагностики гипокальциемии является измерение уровня общего кальция. Корректировка кальция на уровень альбумина крови необходима с целью исключения ложноотрицательных или ложноположительных результатов кальциемии при изменении концентрации плазменных белков.

Формулы для расчета альбумин-скорректированного кальция.

- Общий кальций плазмы (ммоль/л) = измеренный уровень кальция плазмы (ммоль/л) + 0,02 × (40 — измеренный уровень альбумина плазмы (г/л))

- Общий кальций плазмы (мг/дл) = измеренный уровень кальция плазмы (мг/дл) + 0,8 × (4 — измеренный уровень альбумина плазмы (г/дл))

- Коэффициент пересчета: [кальций] мг/дл × 0,25 ==> [кальций] ммоль/л.

Комментарии. Динамический мониторинг общего кальция, альбумина (с расчетом альбумин-скорректированного кальция), фосфора, магния, креатинина с расчетом СКФ в случае компенсации заболевания рекомендовано проводить с частотой 1 раз в 3–6 месяцев. При отсутствии компенсации гипопаратиреоза и/или коррекции доз стандартной терапии рекомендована более частая оценка показателей фосфорно-кальциевого обмена, до нескольких раз в неделю, для оценки адекватности подобранной терапии.

Динамический мониторинг суточной экскреции кальция рекомендовано проводить 1 раз в 6–12 месяцев. В случае выявления гиперкальциурии и/или назначении тиазидов контрольное исследование уровня кальция в суточной моче рекомендовано выполнить через 1,5–2 месяца для оценки адекватности проводимого лечения.

## ИНСТРУМЕНТАЛЬНЫЕ ДИАГНОСТИЧЕСКИЕ ИССЛЕДОВАНИЯ

Комментарии. У пациентов с хроническим гипопаратиреозом на фоне приема терапии препаратами витамина D и его производными и препаратами кальция значимо повышается риск развития нефролитиаза/нефрокальциноза.

Комментарии. Пациентам с длительным анамнезом гипопаратиреоза (более 3–5 лет) показан периодический осмотр у врача-офтальмолога с целью своевременной диагностики развития катаракты и определения потребности в специализированном ее лечении.

Комментарии. Клинические проявления кальцификации различных отделов центральной нервной системы у пациентов с длительным анамнезом гипопаратиреоза неспецифичны. К наиболее распространенным относятся двигательные нарушения: ригидность мышц, паркинсонизм, гиперкинезы (хорея, тремор, дистония, атетоз, орофациальная дискинезия); когнитивные расстройства; мозжечковые симптомы и нарушения речи. В ряде случаев отмечаются эпилептические приступы, деменция. Нередко наблюдается сочетание различных клинических симптомов. Вопрос о наличии патогенетической взаимосвязи между неврологической симптоматикой и объемом, локализацией обызвествлений остается противоречивым. При возникновении данных симптомов и/или выявлении кальцификации базальных ганглиев по данным КТ головного мозга показана консультация врача-невролога.

Комментарии. При гипопаратиреозе костный обмен замедлен, таким образом отсутствуют предпосылки к снижению МПК с течением времени в отсутствие сопутствующих факторов риска, таких как терапия глюкокортикоидами. Для оценки состояния костной ткани пациентам с хроническим гипопаратиреозом желательно проведение комплексного обследования, включающего определение маркеров костного обмена и рентгенографию костей.

## ИНЫЕ ДИАГНОСТИЧЕСКИЕ ИССЛЕДОВАНИЯ

Комментарии:

-пациентам с изолированным гипопаратиреозом неясной этиологии, возникшим после первого года жизни, рекомендуется подробный сбор анамнеза и жалоб пациента, исследование гена AIRE для исключения АПС 1-го типа. Наследственные формы заболевания представлены в таблице 3.

**Table:** Таблица 3. Наследственные формы гипопаратиреоза [40]

Заболевания	Наследование	Локализация	Ген
Синдромальный гипопаратиреоз
Синдром ДиДжорджи 1 типа	AD	22q11.2	TBX1
Синдром ДиДжорджи 2 типа	AD	10p13–14	NEBL
CHARGE синдром	AD	8q12.2	CHD7
АПС 1 типа	AR	21q22.3	AIRE
HDR-синдром	AD	10p15	GATA3
Синдром Кернса–Сейра	Митохондр.	NA	Митохондр. ДНК
Синдром MELAS	Митохондр.	NA	Митохондр. ДНК
Синдром дефицита MTP	AR	2p23	HADHB
Синдром Кенни–Каффи 1 типа	AR	1q42.3	TBCE
Синдром Кенни–Каффи 2 типа	AD	11q12.1	FAM111A
Синдром Саньяд–Сакати	AR	1q42.3	TBCE
Дисплазия «тонких» костей	AD	11q12.1	FAM111A
Аутосомно-доминантная гипокальциемия
Аутосомно-доминантная гипокальциемия 1 типа и синдром Бартера 5 типа	AD	3q21.1	CASR
Аутосомно-доминантная гипокальциемия 2 типа	AD	19p13.3	GNA11
Изолированный гипопаратиреоз
Аутосомный гипопаратиреоз	AD или AR	6p24.2 и 11p15	GCM2 и PTH
Х-связанный гипопаратиреоз	XR	Xq26–27	SOX31

## ЦЕЛИ ЛЕЧЕНИЯ И МОНИТОРИНГА ГИПОПАРАТИРЕОЗА

Основные цели долгосрочной терапии гипопаратиреоза представлены в таблице 4.

**Table:** Таблица 4. Основные цели долгосрочной терапии гипопаратиреоза

Уровень альбумин-скорректированного кальция крови	Поддержание на нижней границе или несколько ниже нижней границы референсного диапазона (2,11–2,65 ммоль/л) у пациентов без клинических симптомов гипокальциемии	2,1−2,3 ммоль/л
Уровень суточной экскреции кальция (исследование уровня кальция в суточной моче)	Поддержание в пределах целевого диапазона	Мужчины: <7,5 ммоль/сут Женщины: <6,25 ммоль/сут
Уровень фосфора сыворотки крови	Поддержание в пределах референсного диапазона	0,8−1,4 ммоль/л
Уровень магния сыворотки крови	В пределах референсного диапазона	0,7−1,05 ммоль/л
Уровень 25(ОН)витамина D	Как в общей популяции	30–60 нг/мл (75–150 нмоль/л)
Общее самочувствие и качество жизни	Персонализированное лечение
Информированность/образование	Информирование пациента о симптомах гипокальциемии и гиперкальциемии, осложнениях заболевания

Комментарии. Терапевтические цели — уровень альбумин-скорректированного кальция крови в пределах 2,1–2,3 ммоль/л или ионизированного кальция в пределах 1,05–1,15 ммоль/л — основаны на поддержании физиологических процессов в организме. Некоторые пациенты могут, однако, нуждаться в более высоких уровнях кальция сыворотки крови для устранения симптомов гипокальциемии.

Комментарии.

- Для больных с гипопаратиреозом, так же как и для общей популяции, в большинстве случаев характерно наличие недостатка или дефицита витамина D. В связи с чем для его коррекции целесообразно использование нативных форм витамина D (колекальциферол**).

- Для российской популяции оптимальные уровни 25(ОН)D установлены в диапазоне 30–60 нг/мл (75–150 нмоль/л). Уровни 25(ОН)D более 100 нг/мл (250 нмоль/л) могут стать причиной токсического воздействия витамина D на организм и не рекомендуются.

- Терапия альфакальцидолом**, кальцитриолом** не оказывает существенного влияния на уровень 25(ОН)D сыворотки крови.

Комментарии. В таблице 6 представлена клиническая симптоматика, о которой следует информировать пациентов, чтобы они могли самостоятельно заподозрить у себя гипо- или гиперкальциемию на ранней стадии (табл. 5).

**Table:** Таблица 5. Клиническая симптоматика, о которой следует информировать пациентов, чтобы они могли самостоятельно заподозрить у себя гипо- или гиперкальциемию на ранней стадии

Органы/системы	Гипокальциемия	Гиперкальциемия
Центральная нервная система	ДепрессияРаздражительностьСпутанность сознания и дезориентацияСудороги	СлабостьГоловная больСонливостьСпутанность сознания и дезориентацияСнижение памяти и концентрации внимания
Нейро-мышечная система	Онемения и покалывания (парестезии) в пальцах рук и ног, околоротовой области	Мышечная слабость
Сердечно-сосудистая система	Частый, аритмичный пульсСимптомы сердечной недостаточности	Частый, аритмичный пульсАртериальная гипертензия
Желудочно-кишечный тракт	Боль в животе	Потеря аппетитаТошнота, рвотаБоль в животеЗапоры
Почки		ПолиурияСухость во рту, жажда
Органы дыхания	Затруднения дыханияСвистящее дыханиеЧувство «сдавления» в горле	

**Table:** Таблица 6. Лекарственные препараты для лечения гипопаратиреоза

Международное непатентованное наименование лекарственного препарата	Средняя суточная доза	Единицы измерения	Кратность приема
Альфакальцидол**	1,0–4,0	мкг	1–3 р/сут
Кальцитриол**	0,25–2,0	мкг	1–3 р/сут
Препараты, содержащие кальция карбонат3	1000–3000	мг	1–4 р/сут
Колекальциферол**	400–8002	МЕ	1 р/сут
Гидрохлортиазид** [80, 88–90]	12,5–100	мг	1–2 р/сут
Хлорталидон1 [90, 91]	50–100	мг	1 р/сут
Комбинации различных солей магния	300–400	мг	1–3 р/сут

## ЛЕЧЕНИЕ ГИПОПАРАТИРЕОЗА

Лекарственные препараты, используемые для лечения гипопаратиреоза представлены в таблице 6.

Комментарии.

- Стандартная терапия гипопаратиреоза включает в себя препараты витамина D и его производные (альфакальцидол**, кальцитриол**) и препараты кальция. Кальциемический эффект кальцитриола** превышает таковой у альфакальцидола** примерно вдвое.

- Для поддержания уровня кальция крови в пределах целевого уровня рекомендуется титрация доз препаратов витамина D и его производных, разделение суммарной дозы препарата в 2–3 приема. Титрация дозы обычно производится с шагом в 0,5 (или 0,25) мкг для альфакальцидола** и 0,25 мкг для кальцитриола**. Больший шаг изменения доз может потребоваться при выраженных гипо/гиперкальциемии. Рекомендуемый временной интервал для коррекции доз препарата витамина D и его производных (альфакальцидол**, кальцитриол**) составляет 2–3 дня, что обусловлено их фармакокинетикой и как следствие, адекватной оценкой проведенных изменений. При малосимптомном течении и умеренных колебаниях показателей кальциемии лабораторная оценка адекватности скорректированных доз может быть произведена через 7–10 дней. Для оценки клинической эффективности подобранной терапии и достижения стабильных значений кальциемии может потребоваться около 2–3 месяцев, особенно для пациентов с высокой потребностью в препаратах витамина D и его производных.

- Изолированное назначение солей кальция патогенетически не оправдано и вызывает лишь кратковременное повышение показателей кальциемии сыворотки крови.

-Пациентам с гипопаратиреозом рекомендуется использовать диету с высоким потреблением кальцийсодержащих продуктов.

- Для лечения гипопаратиреоза используются различные препараты кальция. Как правило применяется препараты, содержащие кальция карбонат (40% элементарного кальция), в том числе в составе комбинированного препарата «кальция карбонат + колекальциферол» в среднесуточных дозах 1–3 г (могут быть использованы и более высокие дозы). Также могут быть использованы пищевые добавки кальция цитрата (21% элементарного кальция). При назначении необходимо учитывать особенности фармакокинетики различных препаратов: кальция карбонат лучше всасывается в кислой среде желудка, поэтому более предпочтителен прием вместе с пищей; кальция цитрат рекомендован пациентам с ахлоргидрией или получающих лечение ингибиторами протонового насоса.

- Высокие дозы препаратов кальция могут снизить потребность в витамине D и его производных и улучшить контроль за поддержанием целевого уровня фосфора сыворотки крови, связывая фосфор в кишечнике.

## ПРОФИЛАКТИКА

Комментарии. Несмотря на то что в настоящее время не разработаны четкие временные критерии для забора крови на ПТГ и показатели кальция в послеоперационном периоде, проведенные исследования демонстрируют ценность измерения данных параметров в течение первых 24 ч после операции на органах шеи. Уровень ПТГ менее 15 пг/мл в первый день после операции рассматривается как предиктор развития послеоперационного гипопаратиреоза (чувствительность 97,7%, специфичность 82,6%). Послеоперационная оценка лабораторных показателей фосфорно-кальциевого обмена — необходимое условие для своевременного назначения препаратов витамина D и его производных и препаратов кальция.

Комментарии. Минимальная рекомендуемая суточная доза пероральных препаратов кальция составляет не менее 3000 мг на срок не менее 2 недель с последующей оценкой показателей фосфорно-кальциевого обмена и определением потребности в продолжении терапии.

Комментарии. К независимым предикторам послеоперационной гипокальциемии относят тиреоидэктомию, особенно с центральной и/или боковой лимфодиссекцией, повторную операцию на органах шеи, интраоперационное повреждение ОЩЖ, а также низкий интра- и послеоперационный уровень ПТГ.

Минимальная рекомендуемая суточная доза пероральных препаратов кальция составляет не менее 3000 мг в сочетании с терапией препаратами витамина D и его производных (#кальцитриол** 1 мкг в сутки) на срок не менее 2 недель с последующей оценкой показателей фосфорно-кальциевого обмена и определением потребности в продолжении терапии [119].

## ЛЕЧЕНИЕ ОСТРОЙ ГИПОКАЛЬЦИЕМИИ

Для купирования острой гипокальциемии рекомендуется установка центрального венозного катетера, что позволит предотвратить склерозирование периферических вен вследствие инфузии кальция. Предпочтительно использование кальция глюконата**, так как кальция хлорид имеет серьезные осложнения в виде некроза мягких тканей, в случае выхода раствора из сосудистого русла.

В зависимости от ситуации могут быть использованы следующие варианты парентерального введения препаратов кальция.

1. Введение кальция глюконата** внутривенно болюсно в количестве 20–60 мл — быстро без разведения 0,9% раствором NaCl** (или в 5% раствора декстрозы). Метод используется при выраженных симптомах гипокальциемии (пациенты с клинической картиной «страха смерти» или в бессознательном состоянии). Доза вводимого кальция определяется по появлению диспепсических жалоб.

2. Введение половины дозы кальция глюконата** внутривенно болюсно без разведения (40–50 мл 10% раствора кальция глюконата**), остальная доза кальция (50–60 мл 10% раствора кальция глюконата**) вводится внутривенно медленно в разведенном состоянии (0,9% раствора NaCl** или 5% декстрозы**) со скоростью для инфузомата 0,5–1,5 мкг/кг/ч. Данный способ введения является самым частым для купирования острой гипокальциемии, позволяющим быстро нормализовать клиническое состояние пациента.

3. Введение всей дозы парентеральных препаратов кальция (80–100 мл 10% раствора кальция глюконата**), разведенного в растворе (0,9% NaCl** или 5% декстрозы**). Метод используется преимущественно для поддержания адекватного уровня кальция с целью профилактики развития острой гипокальциемии.

Парентеральное введение препаратов кальция всегда прекращается при появлении диспептических жалоб (тошнота, рвота).

Внутривенное введение кальция требует осторожности у больных с гипокалиемией и у пациентов, принимающих дигоксин**, в связи с повышенным риском аритмий.

Одновременно назначаются пероральные препараты кальция и препараты витамина D и его производных (альфакальцидол**, кальцитриол**). Цель терапии — купирование симптомов острой гипокальциемии и нормализация показателей общего и ионизированного кальция на нижней границе референсных значений или несколько ниже в отсутствие клинических симптомов гипокальциемии. Для коррекции терапии необходим частый контроль уровня кальция крови (каждые 6–12 ч в начале лечения, после стабилизации состояния пациента — каждые 24 ч).

Стоить отметить, что при наличии у пациента выраженной гипомагниемии показана ее коррекция с использованием как пероральных препаратов (препараты комбинации различных препаратов магния 300–400 мг/сут), так и внутривенных форм — внутривенно струйно 2 г магния сульфата** в течение 10–20 минут, внутривенно капельно 25% раствор магния сульфата** 2–4 г в 150–200 мл физиологического раствора NaCl**.

## ВЕДЕНИЕ БЕРЕМЕННОСТИ ПРИ ГИПОПАРАТИРЕОЗЕ

Рекомендации по ведению беременности у пациентки с хроническим гипопаратиреозом:

## ЗАКЛЮЧЕНИЕ

Гипопаратиреоз является относительно редким эндокринным заболеванием, требующим многокомпонентной лекарственной терапии и тщательного наблюдения для контроля над заболеванием и снижения рисков развития осложнений. Создание клинических рекомендаций — необходимая инициатива, направленная на улучшение качества оказания медицинской помощи пациентам с данной нозологией.

## ПРИЛОЖЕНИЕ А. МЕТОДОЛОГИЯ РАЗРАБОТКИ КЛИНИЧЕСКИХ РЕКОМЕНДАЦИЙ

**Table:** Таблица 1. Шкала оценки уровней достоверности доказательств (УДД) для методов диагностики (диагностических вмешательств)

УДД	Расшифровка
1	Систематические обзоры исследований с контролем референсным методом или систематический обзор рандомизированных клинических исследований с применением метаанализа
2	Отдельные исследования с контролем референсным методом или отдельные рандомизированные клинические исследования и систематические обзоры исследований любого дизайна, за исключением рандомизированных клинических исследований, с применением метаанализа
3	Исследования без последовательного контроля референсным методом или исследования с референсным методом, не являющимся независимым от исследуемого метода или нерандомизированные сравнительные исследования, в том числе когортные исследования
4	Несравнительные исследования, описание клинического случая
5	Имеется лишь обоснование механизма действия или мнение экспертов

 

**Table:** Таблица 2. Шкала оценки уровней достоверности доказательств (УДД) для методов профилактики, лечения и реабилитации (профилактических, лечебных, реабилитационных вмешательств)

УДД	Расшифровка
1	Систематический обзор РКИ с применением метаанализа
2	Отдельные РКИ и систематические обзоры исследований любого дизайна, за исключением РКИ, с применением метаанализа
3	Нерандомизированные сравнительные исследования, в т.ч. когортные исследования
4	Несравнительные исследования, описание клинического случая или серии случаев, исследования «случай-контроль»
5	Имеется лишь обоснование механизма действия вмешательства (доклинические исследования) или мнение экспертов

**Table:** Таблица 3. Шкала оценки уровней убедительности рекомендаций (УУР) для методов профилактики, диагностики, лечения и реабилитации (профилактических, диагностических, лечебных, реабилитационных вмешательств)

УУР	Расшифровка
A	Сильная рекомендация (все рассматриваемые критерии эффективности (исходы) являются важными, все исследования имеют высокое или удовлетворительное методологическое качество, их выводы по интересующим исходам являются согласованными)
B	Условная рекомендация (не все рассматриваемые критерии эффективности (исходы) являются важными, не все исследования имеют высокое или удовлетворительное методологическое качество и/или их выводы по интересующим исходам не являются согласованными)
C	Слабая рекомендация (отсутствие доказательств надлежащего качества (все рассматриваемые критерии эффективности (исходы) являются неважными, все исследования имеют низкое методологическое качество и их выводы по интересующим исходам не являются согласованными)

## ПРИЛОЖЕНИЕ Б. АЛГОРИТМЫ ДЕЙСТВИЙ ВРАЧА

**Figure:**
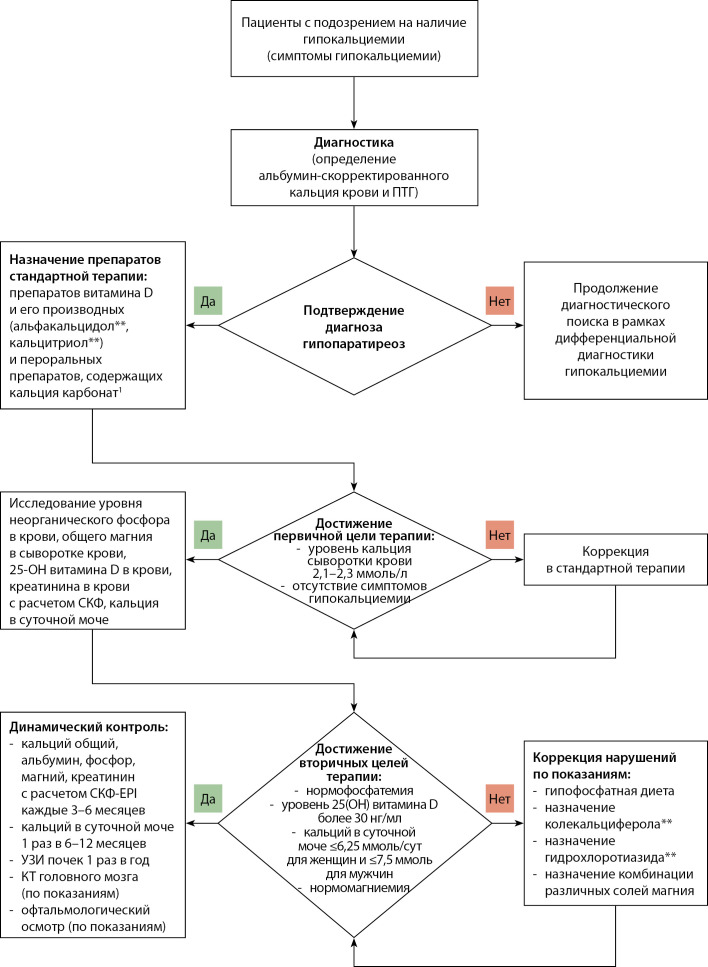
1 — в том числе в составе комбинированного препарата «кальция карбонат + колекальциферол»
